# The unequal vulnerability of communities of color to wildfire

**DOI:** 10.1371/journal.pone.0205825

**Published:** 2018-11-02

**Authors:** Ian P. Davies, Ryan D. Haugo, James C. Robertson, Phillip S. Levin

**Affiliations:** 1 School of Environmental and Forest Sciences, University of Washington, Seattle, Washington, United States of America; 2 The Nature Conservancy, Portland, Oregon, United States of America; 3 The Nature Conservancy, Seattle, Washington, United States of America; Oregon State University, UNITED STATES

## Abstract

Globally, environmental disasters impact billions of people and cost trillions of dollars in damage, and their impacts are often felt most acutely by minority and poor communities. Wildfires in the U.S. have similarly outsized impacts on vulnerable communities, though the ethnic and geographic distribution of those communities may be different than for other hazards. Here, we develop a social-ecological approach for characterizing fire vulnerability and apply it to >70,000 census tracts across the United States. Our approach incorporates both the wildfire potential of a landscape and socioeconomic attributes of overlying communities. We find that over 29 million Americans live with significant potential for extreme wildfires, a majority of whom are white and socioeconomically secure. Within this segment, however, are 12 million socially vulnerable Americans for whom a wildfire event could be devastating. Additionally, wildfire vulnerability is spread unequally across race and ethnicity, with census tracts that were majority Black, Hispanic or Native American experiencing ca. 50% greater vulnerability to wildfire compared to other census tracts. Embracing a social-ecological perspective of fire-prone landscapes allows for the identification of areas that are poorly equipped to respond to wildfires.

## Introduction

People living in low-income countries and poor people living in affluent countries tend to suffer disproportionately from environmental disasters. The last two decades saw over 7,000 major environmental disasters that caused trillions of dollars in damage and killed more than 1.35 million people worldwide [1]. In this same time period, more than three times the number of people died per disaster in low-income countries than in high-income countries [1]. Even within countries that are more affluent and experience fewer disasters, the impacts of those disasters that do occur can be strikingly unequal. For instance, when Hurricane Katrina struck New Orleans, the impacts to life and property were disproportionately borne by the African American community–damaged areas comprised 46% Black people versus 26% in undamaged areas, [2] and 84% of missing people were Black in a city that is only 68% Black [3]. When it comes to disasters, differences in vulnerability can affect the magnitude and duration of impacts like the loss of property, livelihoods, or services.

A widespread understanding of environmental disasters is that they are beyond human control–hurricanes occur to unsuspecting coastal communities, earthquakes strike without warning, and so on. In recent decades however, scholars from a range of disciplines have argued that natural disasters are not “natural” [4,5]. Rather, it is the social, political, and economic context that makes an environmental hazard become a disaster [6,7]. Just as a hurricane can leave one city in ruin for years to come while a similar storm leaves another city unscathed, so too do various human factors generate unequal exposure and susceptibility to wildfires. For example, a strategy to aggressively suppress wildfires near homes promotes increased intensity of wildfires in the future due to fuel accumulation [8–10]. At a household level, families without financial means cannot afford tree trimming, brush removal, or other fire mitigation services that could mean the difference between a low severity under-burn and a severe wildfire [11]. Additionally, families who rent are ineligible for much of the federal assistance available to homeowners for rebuilding after a fire event. [12]. In these ways, a sole focus on biophysical wildfire hazards like fuel and weather conceals the root causes that turn fire, a natural process, into a disaster.

Geographic segregation of fire-prone places in the U.S. does not operate identically to other hazard-prone areas around the world. In developing countries, poor and ethnic minorities are more likely to live in hazardous areas, like flood-prone farmland in India or volcanically-active slopes in Guatemala [13,14]. This is not so in the U.S., where incentives like response aid, environmental amenities, and wildfire insurance facilitate the settlement of economically advantaged groups in fire-prone landscapes, oftentimes in second homes [14,15]. This seems to complicate traditional disaster narratives associating place-based hazards with poverty, but the important point is not just that socially vulnerable populations may be more *exposed* to environmental hazards, but that those hazards become disasters specifically when they affect vulnerable populations.

Fire is a basic, necessary, and unavoidable component of many landscapes and underlies the delivery of a number of key ecosystem services in forests and rangelands around the globe [16]. Even so, wildfires since 1984 have affected nearly 6 million people, directly caused over 1,900 deaths, and generated more than $52 billion in economic costs [17]. While fire-prone places in the U.S. are more likely to be populated by higher-income groups, this fact threatens to overshadow the thousands of low-income individuals who also live in fire-prone places but lack the resources to prepare or recover from fire [15]. In California, for example, many individuals in rural areas, low-income neighborhoods, and immigrant communities do not have access to the resources necessary to pay for insurance, rebuilding, or continual investment in fire safety, thereby increasing their vulnerability to wildfire [18]. These disparities became very clear after the 2017 wildfires in Sonoma County, California, where price gouging on rentals worsened an already dire housing shortage [19].

Conceptualizing wildfire disasters as the product of the complete social-ecological system can provide novel insights into management. By understanding who is vulnerable and why, management can expand beyond technical fixes to socioeconomic and political solutions. Here we use a vulnerability assessment framework to identify places across the coterminous United States that are disproportionately imperiled by wildfire. This study can be seen as building on wildfire vulnerability scholarship by Wigtil et al. (2016), Collins (2012), and others while explicitly exploring the relationships between race, geography, and wildfire from an environmental justice perspective [14,15].

## Methods

### Overview of approach

**Fig 1:**
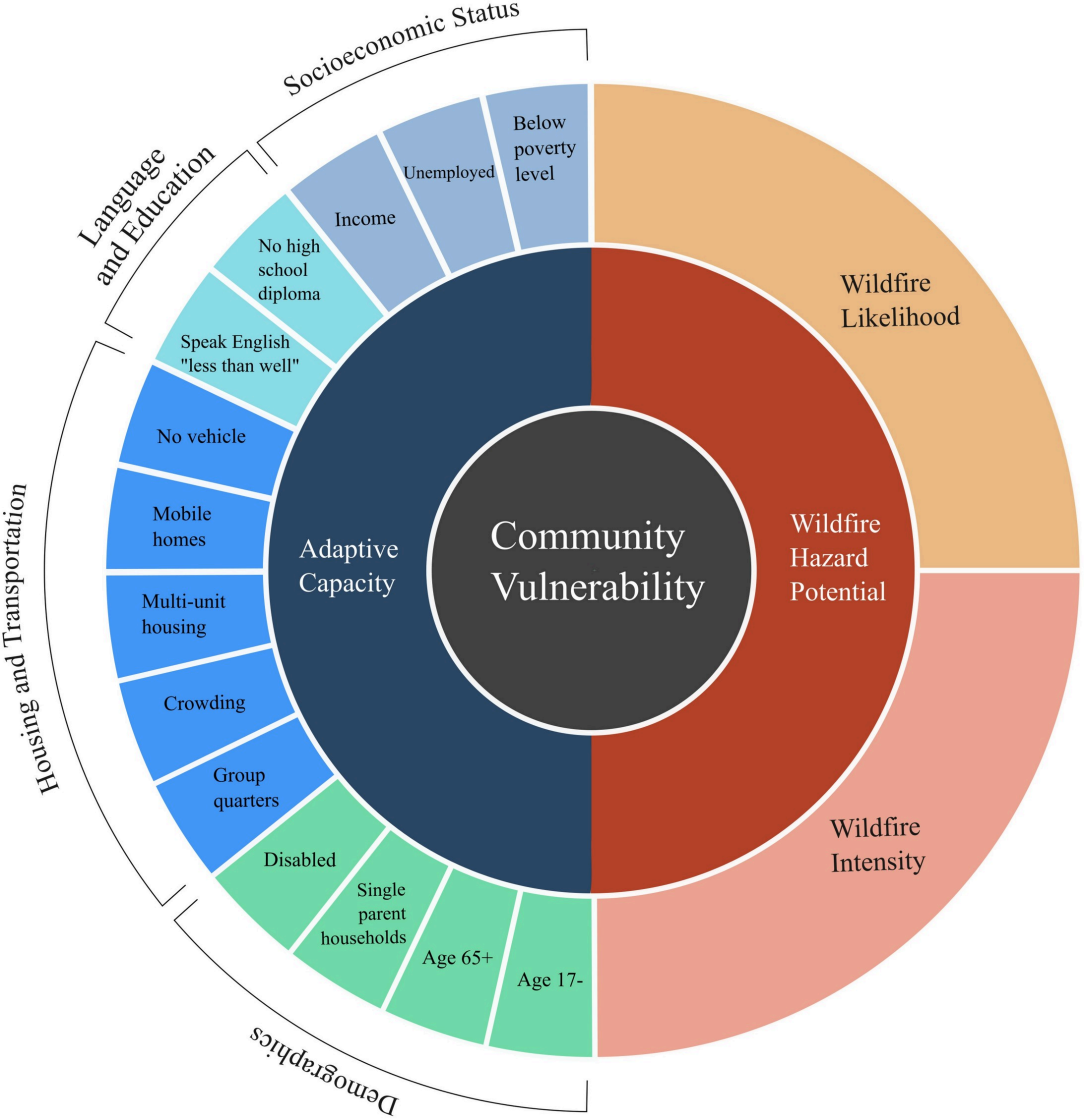
This wildfire vulnerability framework reflects both the potential of wildfire and the adaptive capacity of a census tract. Outer wedges represent the respective components of wildfire hazard potential and census tract adaptive capacity, though the size of the wedges does not correspond to any weighting scheme.

We use a vulnerability assessment framework to identify census tracts that are disproportionately imperiled by wildfire (Fig 1). We define wildfire vulnerability as a combination of the exposure of social-ecological systems to a hazard, such as wildfire, and the adaptive capacity of a place to absorb, recover, and modify exposure to the hazard [6,20]. This framework captures both the potential of wildfire as well as the socioeconomic adaptive capacity of census tracts exposed to wildfire.

### Estimating U.S. fire vulnerability

#### Wildfire hazard potential

We analyzed the potential of wildfire and adaptive capacity for each of the 71,901 U.S. census tracts (2014). The United States Forest Service (USFS) estimated Wildfire Hazard Potential (WHP) for the continental U.S [21]. The WHP is a 270-meter resolution raster product derived from the Large Fire Simulator [22], incorporating fuels, vegetation, weather, historical fire occurrence, and resistance to control data to estimate the probability that an area would experience extreme fire behavior under conducive weather conditions [21]. The WHP values are binned such that two-thirds of U.S. land area is classified as having low or very low wildfire potential and only one-third classified as having moderate, high, or very high wildfire potential. We rescaled WHP in this analysis from 0–1 to match the adaptive capacity index (described below), with 0 being unburnable land and 1 being very high potential for wildfire that is difficult to control. Higher WHP values correspond to higher potential for more extreme wildfires. We then estimated the WHP for each census tract as the average of underlying WHP raster cells. Vulnerability to fire requires at least a minimal exposure to wildfire, so for the purpose of this analysis we constrained our estimates to the 6,304 census tracts that have at least moderate fire potential. This effectively eliminates the most urbanized areas that lack fuels and other conditions necessary for wildfire.

#### Census tract adaptive capacity

We next estimated the adaptive capacity of each census tract using data from the 2014 U.S. Census American Community Survey. Census data are an imperfect but commonly employed proxy for communities and neighborhoods. They are imperfect because neighborhoods and communities are socially-constructed based on both shared meaning and geography which are difficult to capture (although the census does consider both socioeconomic and demographic characteristics when delineating tracts) [23]. Despite this, census data are ubiquitous in the social science literature because of their coverage and availability [23]. We employ census tracts as the unit of analysis in this study because they are available at the national level and are groupings at which wildfire policy and management are often prescribed and implemented. With that in mind, we acknowledge that local results may differ with alternative geographies.

By adaptive capacity, we are referring to the ability of a census tract to absorb and adjust to disturbances, like wildfire, while minimizing damage to life, property, and services [6,24]. The ability to adapt to hazards is influenced by a number of social and demographic factors, including age, income, the strength of social networks, and neighborhood characteristics [25]. For example, economically disadvantaged families, the elderly, disabled people, and residents of high-rise apartments or mobile homes tend to be less adaptable to hazards [26]. The least adaptable groups are likely those whose needs are insufficiently considered in the planning of local response and relief organizations [27].

Following Flanagan and colleagues, we created an adaptive capacity index based on 13 metrics from the U.S. Census American Community Survey [25]. The index was comprised of four domains: socioeconomic status, language and education, demographics, and housing and transportation (S1 Table).

Socioeconomic status was characterized with three metrics: Persons below poverty level, the number of people (age 16+) unemployed, and per capita income. Poor households often cannot afford to pay for fire mitigation services like tree cutting and removal of fine fuels [11]. While wealthier residents may be disinclined to remove environmental amenities like trees, they are more likely to have fire insurance and the community firefighting resources needed to extinguish a fire [11,28]. Additionally, lower income households face more obstacles rebuilding or finding new housing after a fire [29].

Higher educational level also appears to improve adaptive capacity. In general, education improves access to relevant information, enlarging social networks that can facilitate recovery, and aiding in the navigation of bureaucratic hurdles [25,30]. Limited proficiency in English has also been linked to difficulty recovering from disasters [25,26].

Housing quality and transportation both covary with wealth, with economically disadvantaged people often living in poorly constructed housing or mobile homes [31]. Multi-unit housing (apartments, group facilities, farm worker dormitories, etc.) poses an increased risk to health as escape routes can be overcrowded and building-owners are less likely to pursue fire mitigation on their properties [18,32]. Additionally, renters are eligible for less federal housing assistance than homeowners [12]. Finally, transportation out of an evacuation zone may be challenging for those without access to a vehicle, and for some, fuel costs may prevent vehicle use [33].

To construct the adaptive capacity index, we adopted the framework of Flanagan and colleagues [25]. Briefly, we ranked all 71,901 census tracts across each of the 13 indicators such that each census tract had 13 rankings from 1 to 71,901. We then converted these ranks to percentile ranks from 1 to 100, summed across the 13 values for each census tract, and then calculated the percent rank of those sums. The result is an index from 0–1 for each census tract, *with 0 being those with the greatest capacity to adapt to a wildfire* and 1 being those with the least. That lower numbers of the index correspond to greater adaptive capacity is unintuitive, but this was done because the census variables we use (Fig 1) capture inherently negative properties that worsen as the derived index increases. To combine this intuitively with WHP, which denotes greater fire potential at higher values, we retain this scale so that a higher number denotes a census tract with lower adaptive capacity.

#### Estimating census tract vulnerability

In the risk assessment literature, wildfire vulnerability has been defined as the combination of wildfire likelihood and intensity in a particular place (exposure) with the propensity of that place to experience a change in value (susceptibility) [34]. This is a purely biophysical perspective of vulnerability which does not incorporate the socioeconomic factors responsible for turning wildfire events into wildfire disasters. Here, we adopt a definition of vulnerability from the disaster and hazard literature that also incorporates the social characteristics of affected inhabitants [35]. Following this scholarship, we define the relative vulnerability *V* to wildfire of a census tract as the Euclidean distance of the census tract to the graphical origin (0, 0) in a space defined by the WHP and adaptive capacity index (AC)[36], or

$$V=\sqrt{{\left(AC-{AC}_{min}\right)}^{2}+{\left(WHP-{WHP}_{min}\right)}^{2}}$$

Under this framework, the WHP and adaptive capacity received equal weight and the vulnerability of a census tract increases with distance from the origin of the plot.

### Analysis

We examined the relationship between vulnerability to wildfire and ethnicity in census tracts using quantile regression. Simple linear regression neglects change in the relationship at the edges of the distribution. This is particularly important in situations where some factor acts as a constraint on a variable. In this case, the estimated effects of a factor will not be well represented by changes in the mean of the response variable distribution. Because a number of historical, economic, and social issues may constrain the relationship between wildfire vulnerability and ethnicity we used quantile regression from the R package *quantreg* [37] to explore race and wildfire vulnerability (48). Quantile regression performs regression on subsets of the data divided at different levels (i.e. quantiles) and estimates the conditional quantile *Q*^*τ*^ given the predictor variable. Thus for two quantiles, τ = 0.05, 0.95, we estimate two equations for each racial/ethnic group of the standard form

$$Q^{\tau }(x_{i}|{WHP}_{i})={\beta^{\tau }}_{0}+{\beta^{\tau }}_{1}x_{i}$$

where *x*_*i*_ is the population of a racial/ethnic group *i* in a census tract. The regression yields the 95^th^ and 5^th^ quantile weights for racial and ethnic populations given a wildfire vulnerability in a census tract.

All spatial visualizations and analyses were conducted with ArcGIS v10.4 and R v3.3.2.

## Results

**Fig 2:**
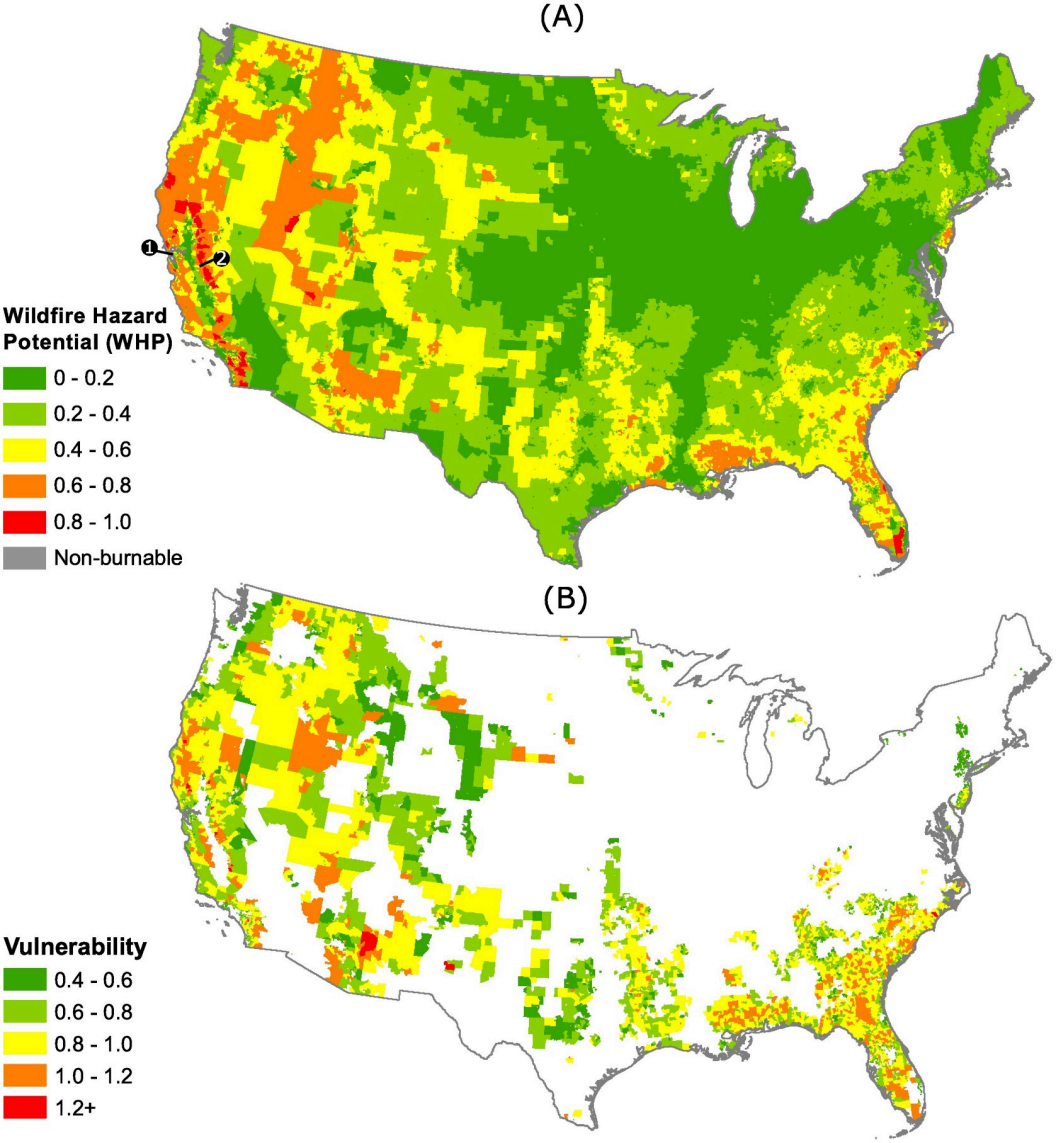
**(A)** Average WHP scores for census tracts in the continental U.S. (n = 71,901). The relative magnitude shifts when we consider the social and economic characteristics of census tracts in their vulnerability to wildfire **(B)**. Only those census tracts with a moderate to very high WHP score are represented in (n = 6,304). In 2A, the San Francisco Bay Area suburbs and eastern Sierra Nevada Mountain communities are labeled as 1 and 2, respectively.

The potential for high-intensity wildfires is spatially heterogeneous, with higher potential in the U.S. west and southeast (Fig 2A and 2B).

**Fig 3:**
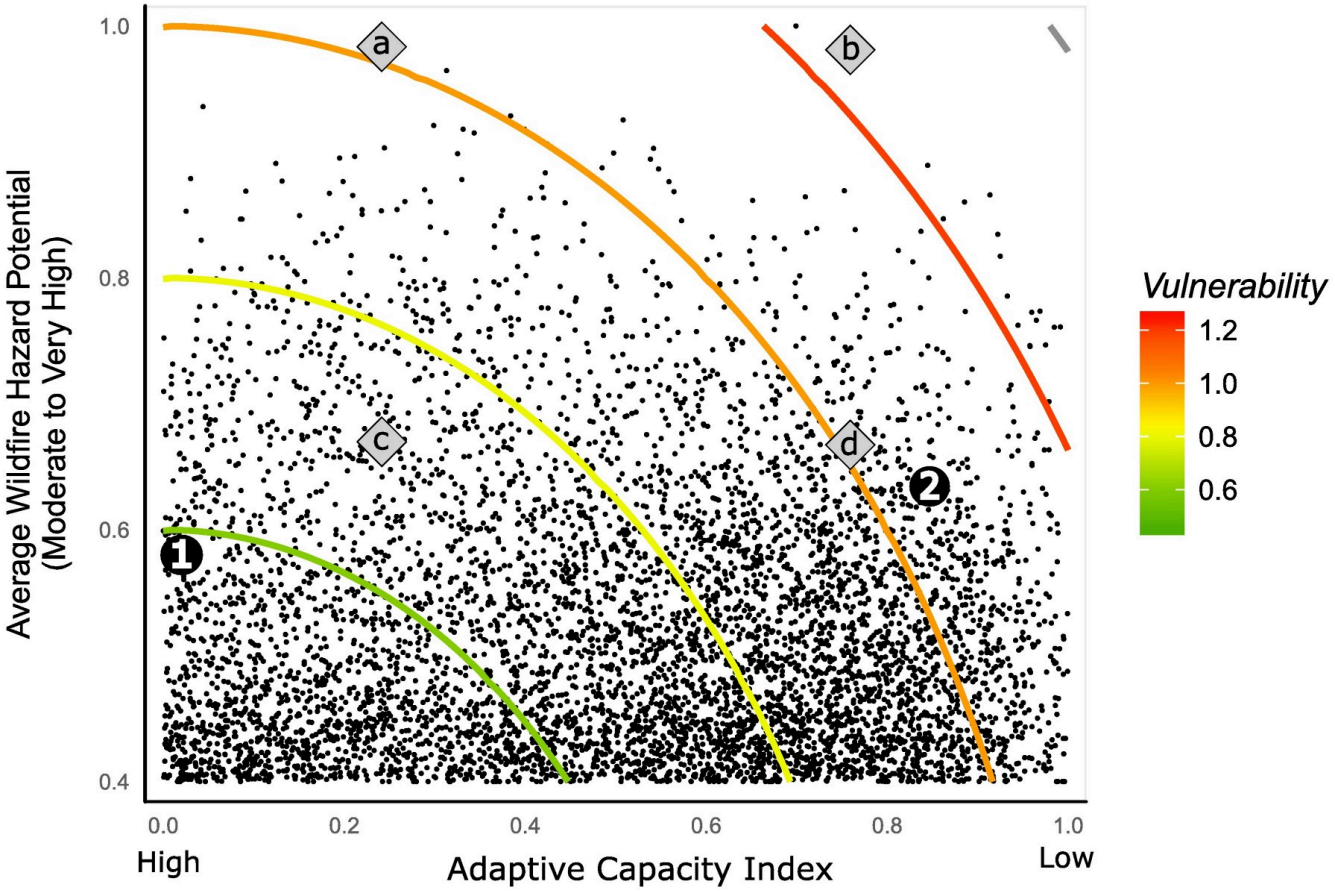
Fire vulnerability for 6,304 U.S. census tracts with moderate to very high WHP. Both the fire potential of the surrounding landscape and the adaptive capacity of a census tract receive equal weight in constituting vulnerability to fire. This vulnerability increases with distance from the origin. The adaptive capacity—WHP space can be divided into four quadrants representing varying levels of adaptive capacity and wildfire hazard: (A) high fire potential—low adaptive capacity; (B) high fire potential—high adaptive capacity; (C) moderate fire potential—low adaptive capacity; (D) moderate fire potential—high adaptive capacity. Americans within the quadrant of moderate fire potential—high adaptive deficit (C) are the least vulnerable, while those with high fire potential—low adaptive deficit (B) are the most vulnerable (note, ‘low’ and ‘high’ adaptive capacity refer to the characteristics captured by the index, not the actual low and high values of the index which are reversed so that it matches intuitively with WHP). Like Fig 2A, the average values of the San Francisco Bay Area suburbs and eastern Sierra Nevada Mountain communities are labeled as 1 and 2, respectively. Note that while these areas have similar WHP, dramatically different adaptive deficits put them in different quadrants.

However, by incorporating adaptive capacity into our estimation of the vulnerability of communities to wildfire (Figs 2B and 3) we generate a different perspective of the threat of wildfire. For instance, if we consider only WHP, the Southeastern U.S. generally exhibits moderate scores, with few places having very high potential for extreme wildfires. In contrast, when we consider the threat of wildfire from a social-ecological perspective, the Southeastern U.S. stands out as a region of high vulnerability. At a smaller-scale, similar shifts are evident: affluent exurban regions east of the San Francisco Bay and rural areas of the eastern Sierra Nevada Mountains in California have similar wildfire potential, but relatively poorer socioeconomic conditions in the Sierra Nevada Mountains make those communities far more vulnerable to fire disaster than their exurban counterparts. These areas are labeled in both Figs 2A and 3.

**Fig 4:**
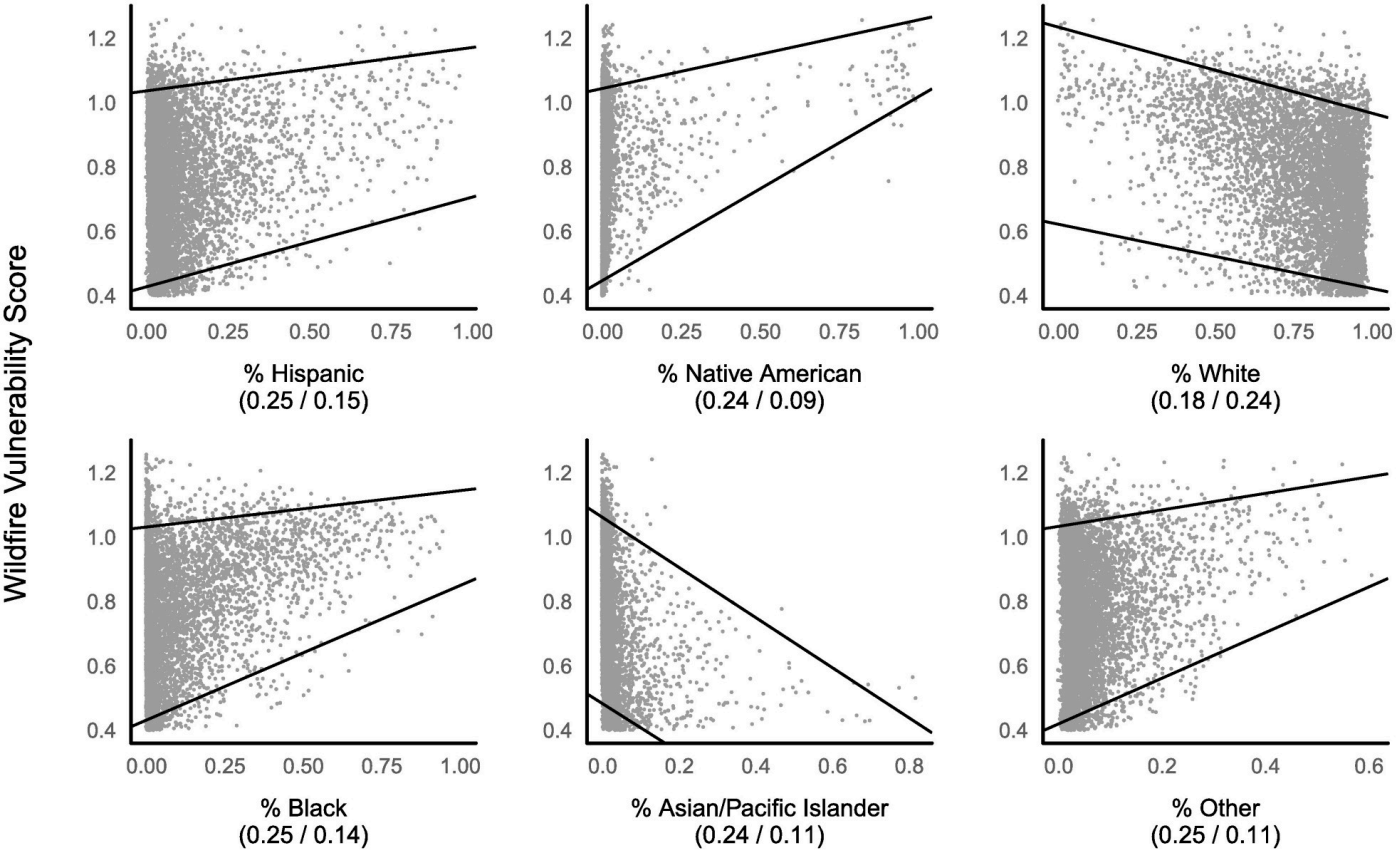
Vulnerability versus race/ethnicity for U.S. census tracts with moderate to very high WHP. Regression lines are calculated at the 5^th^ and 95^th^ vulnerability quantiles, respectively. Vulnerability coefficients of variation (CV) for census tracts with the lowest/highest proportions of a particular race/ethnicity (≤ minimum + 25% and ≥ maximum—25%, respectively) are noted below each plots. Race “Other” includes those who identify as two or more races or a race not listed on the census form. As the proportion of Hispanics, Native Americans, Blacks, or Other increases in a census tract, the range of possible vulnerability scores for that tract becomes restricted to high values. Strikingly, vulnerability actually decreases as the proportion of White and Asian/Pacific Islanders increases.

When we examine the association between wildfire vulnerability and race, quantile regression analyses reveal that some ethnic groups experience very different vulnerability to wildfire than majority-white communities (Fig 4).

In particular, the minimum vulnerability to wildfire experienced by communities increases as the proportion of Native Americans and Blacks increases (p < 0.01 for all groups, see S4 Table for coefficients). A similar trend occurs in Hispanic communities. In contrast, as the proportion of Whites and Asians/Pacific Islanders increases, the minimum wildfire vulnerability that these communities experience declines. Consequently, census tracts experience an upwards compression of the minimum possible vulnerability score as they become more Hispanic, Black, or Native American, while the opposite is true as the proportion of Whites or Asians/Pacific Islanders increases.

**Fig 5:**
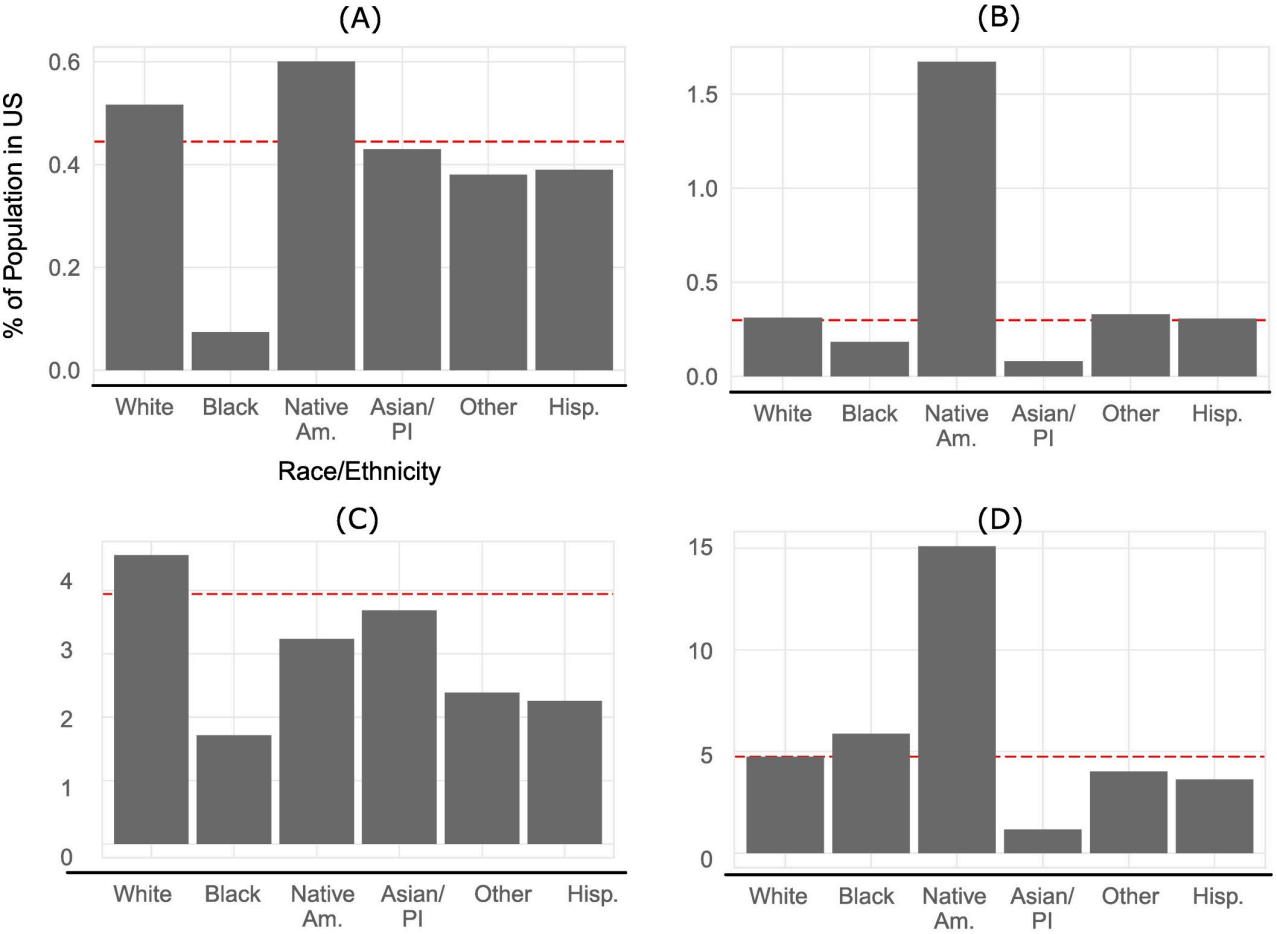
Race/Ethnicity across different quadrants of vulnerability. Values are expressed as the ratio between the population of each race/ethnicity that lives in a quadrant and the total U.S. population of that race/ethnicity. The dotted red line corresponds to the expected share of the population if all groups were equally distributed across the United States. Native Americans are highly overrepresented in the most vulnerable areas (A, B, D) while all non-white populations are underrepresented in the least vulnerable areas (C). In general, white Americans live in areas that are prone to wildfire in greater numbers than would be expected, but these communities are often resilient and better able to respond and adapt to fires.

We divided our estimates of wildfire vulnerability shown in Fig 3 into four quadrants: (A) high fire potential—low adaptive deficit, (B) high fire potential—high adaptive deficit, (C) moderate fire potential—low adaptive deficit, (D) moderate fire potential—high adaptive deficit, and examined the racial composition of each of these quadrants (Fig 5).

All non-white populations are underrepresented in the most secure census tracts. This quadrant (Fig 5C), with moderate fire potential but a low adaptive capacity and thus greater ability to respond to wildfires, is the most populous in our analysis with nearly 12.1 million Americans. Nearly 1.4 million people live in communities that are more prone to fires but have a similarly low adaptive capacity (Fig 5A). While Blacks do not tend to live in the areas with the highest fire potential, they are overrepresented in communities somewhat prone to wildfire but that would likely not respond or adapt well if one were to occur (Fig 5D). Strikingly, Native Americans are highly overrepresented in all of the most vulnerable areas, especially communities with extreme fire potential but high adaptive capacity where they are nearly six times more likely to live than is expected (Fig 5B).

## Discussion

Nearly 29 million Americans live in census tracts with a moderate to very high potential for high-intensity wildfires. White Americans, who constitute 72% of the United States, make up 76% of these census tracts and the population is not, for the most part, socially vulnerable. This is congruent with other research that suggests that environmental amenities and fire insurance facilitate the settlement of more advantaged families in these areas [14,15]. However, an emphasis on only the fringes of fire hazard neglects the 12.4 million people living in census tracts with poor adaptive capacity and lower, but still significant, potential for wildfires. We argue that inhabitants with the lowest adaptability even in these moderately fire-prone landscapes are particularly vulnerable to wildfire and should be central to our understanding of fire disasters.

There is some correlation between social vulnerability and race/ethnicity (S1 Table), and lower real-estate prices in some fire-prone areas may help explain higher numbers of socially vulnerable populations in these locations [38,39]. But it is not just economics–historical patterns of settlement, displacement, and migration have led to the spatial distribution of racial and ethnic groups in the U.S. that we see today [40]. For example, a likely constraint on the upward compression of wildfire vulnerability for Native Americans is their historical forced concentration on federal Indian reservations. Census tribal tracts in these reservations, particularly in the western U.S., have higher WHP scores on average than non-reservation census tracts (S2 Fig). This elevated wildfire potential in tandem with lower adaptive capacity makes Native Americans particularly vulnerable to wildfire disasters.

Differences between the spatial pattern of wildfire hazard potential and vulnerability are due, in part, to the relative scarcity of locations that have high or very high wildfire potential–only 2% of census tracts (n = 1,416) have a WHP ≥ 3. Consequently, the inclusion of socioeconomic, education, housing, and transportation metrics results in the elevation of locations with relatively moderate wildfire potential, but high adaptive deficit. It is also worth noting that by constraining our analysis to only census tracts with moderate to very high WHP, we largely excluded urban areas. Thus, our subset contains less than 7% of the U.S. Black population but over 20% of the U.S. Native American population–groups that, for historical reasons, dwell primarily in urban and rural areas, respectively. A presently unstudied question is how wildfire vulnerability will change as the geography of poverty shifts from cities with low wildfire potential to suburbs and exurbs that border the wildland-urban interface [41].

Emergency planning and mitigation strategies must be tailored to the diverse populations affected by fire, yet the engagement of socially vulnerable groups, particularly of non-white Americans, is still quite limited [42]. Indeed, in 2014, as a massive fire emerged in eastern Washington, language barriers prevented Hispanic farm-workers from receiving evacuation notification from authorities, and the only Spanish radio station in this region never received the emergency information [43]. Similarly, emergency departments and radio stations in Northern California and Santa Barbara struggled to release timely and correct bilingual information during the 2017 wildfires [44]. While this study does not directly assess multilingual evacuation warnings across the U.S., the vulnerability index does incorporate English-speaking ability and ethnicity. These results help make the case that correctly translating and effectively disseminating preparedness and evacuation materials is a prerequisite for equitably mitigating wildfire vulnerability.

Cultural, historical or political experiences differ amongst racial and ethnic populations and these, in turn, also affect preferences for fire management of different communities. For example, in Washington, when the U.S. National Guard was deployed to provide emergency assistance, undocumented migrant farmworkers viewed them as government authorities and threats rather than as trusted helpers and messengers [45]. Similarly, lower than average trust in government by black Americans may underlie substantially greater reluctance towards fire mitigation practices than exhibited by white communities [46]. In contrast, many Native American cultures used fire to maintain or even enhance the value of landscapes. Indeed, many culturally important species require low-intensity fires, while recent severe wildfires resulting from suppression have had a detrimental impact on Native American cultural attributes [8].

We found that Whites and Asian/Pacific Islanders are less likely to live in the census tracts most vulnerable to wildfire. While the number of White residents in a census tract is correlated with WHP, it is negatively correlated with the adaptive capacity index, suggesting White Americans live in areas with better adaptive capacity (S3 and S4 Figs). In other words, the Whites living in the WUI tend to have fewer of the marks of vulnerability than other ethnic groups living there. Asian/Pacific Islanders tend to live in neither fire-prone places nor places with a high adaptive deficit, settling instead in metropolitan areas or more affluent suburbs. However, this is a coarse census category with much intra-group variation, so it is possible that less affluent ethnic groups like Southeast Asians may be more vulnerable to wildfires occurring in places that both border the WUI and contain large Asian/Pacific Islander populations, like Southern California.

Environmental justice issues tied to wildfire extend beyond property destruction and loss of livelihood. For example, smoke from wildfires has the potential to cause a significant health burden on nearby populations. A handful of studies on smoke inhalation from wildfires in Australia found divergent health outcomes between Aboriginal and non-Aboriginal populations, but there is little to no research on such racial disparities in the U.S. While this is an important topic, the wide geographic range of smoke exposure (with effects found at 200–300 miles from wildfires) would likely require a different approach than our method of using census tracts or other small geographies [47]. Another question for further research is how we might increase the adaptive capacity of communities vulnerable to wildfire. Educational programs exist to create more fire-adapted communities, and some county and state agencies have cost-sharing programs to assist homeowners with reducing fuels on their properties. Whether these programs are reducing wildfire potential in the most vulnerable communities is an important question; one study found limited involvement of socially vulnerable populations in federal programs in Arizona, but more research remains to be done in other communities [42].

Wildfire disasters, which disproportionately disrupt the lives of the most socioeconomically disadvantaged, are as much products of social circumstances as they are ecological ones. The number of wildfires that are difficult to suppress is likely to increase as the climate becomes warmer and drier, wildland-urban interfaces see more development, and fuels continue to accumulate [48]. Therefore, wildfire management and alleviation of the factors that influence social vulnerability must be pursued in tandem to reduce the vulnerability communities to wildfires. Embracing a social-ecological perspective of fire-prone landscapes requires more than just accepting that fires will occur [35]—it forces us to consider variability in the capacity of communities to recovery from disturbance [49], cultural differences and experiences with wildfire [50], and disparate histories of exposure to wildfire. Approaching wildfire adaptation from this social-ecological perspective is a first step in creating safer, just, and more resilient communities.

## Supporting information

S1 Fig Census tracts divided into vulnerability quadrants. High fire potential–high low adaptive capacity (A), high fire potential–high adaptive capacity (B), moderate fire potential–low adaptive capacity (C), and moderate fire potential–high adaptive capacity (D).
(PDF)

S2 Fig Federal Indian reservations and moderate to very high WHP. Pictured here is the original WHP raster, not the census tract average used in the analysis of the paper.
(PDF)

S3 Fig Race/Ethnicity vs. WHP for census tracts with moderate to very high WHP. (PDF)

S4 Fig Race/Ethnicity vs. adaptive capacity for census tracts with moderate to very high WHP. (PDF)

S1 Table Adaptive capacity index variables. Variables adapted from the Social Vulnerability Index using data from the American Community Survey 2000–2014 5-year estimates.
(DOCX)

S2 Table Correlation between race/ethnicity in census tracts and adaptive capacity. (DOCX)

S3 Table Correlation between race/ethnicity in census tracts and WHP. (DOCX)

S4 Table Slope (β) and standard error (SE) of quantile regression coefficients at 5th and 95th quantiles. A greater β at the 0.5 vulnerability quantile indicates that increases in the share of Hispanics, Blacks, Native Americans, and Other races is associated with a greater increase in vulnerability for the least vulnerable census tracts than for the most vulnerable tracts. In other words, increasing the population of these groups leads to more dramatic jumps in vulnerability for initially less vulnerable tracts.
(DOCX)

S1 Data Data used for the analysis. (ZIP)
