# Supplementary figures and images for: The unequal vulnerability of communities of color to wildfire
Source: PLoS One. 2018 Nov 2;13(11):e0205825. doi: 10.1371/journal.pone.0205825 (PMC6214520; doi:10.1371/journal.pone.0205825)

(A)

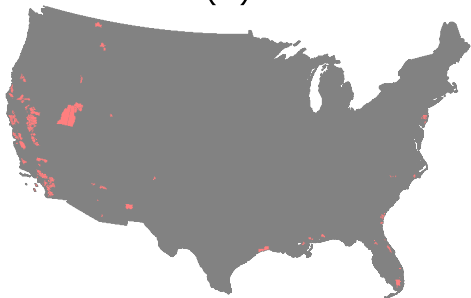

(B)

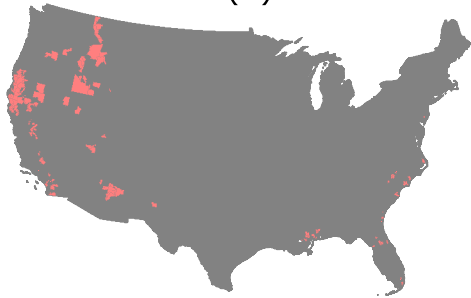

(C)

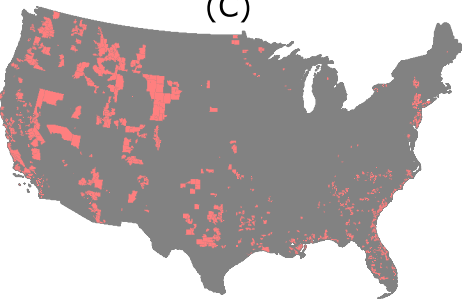

(D)

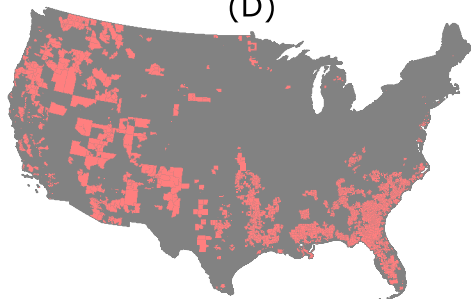

Supplement: S1 Fig — High fire potential–high low adaptive capacity (A), high fire potential–high adaptive capacity (B), moderate fire potential–low adaptive capacity (C), and moderate fire potential–high adaptive capacity (D). (PDF) [file pone.0205825.s001.pdf]

### Wildfire Hazard Potential (WHP)

Moderate

High

Very High

Federal Indian Reservations

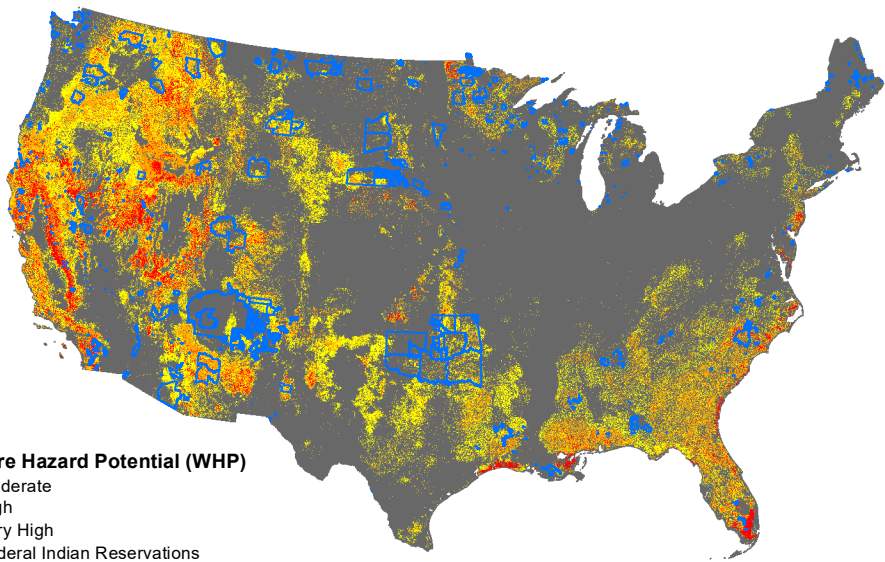

Supplement: S2 Fig — Pictured here is the original WHP raster, not the census tract average used in the analysis of the paper. (PDF) [file pone.0205825.s002.pdf]

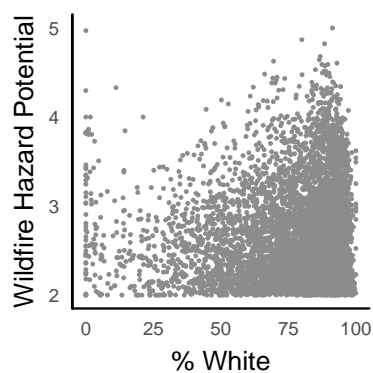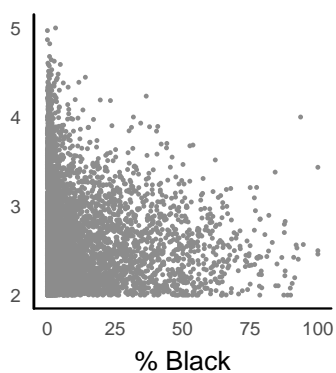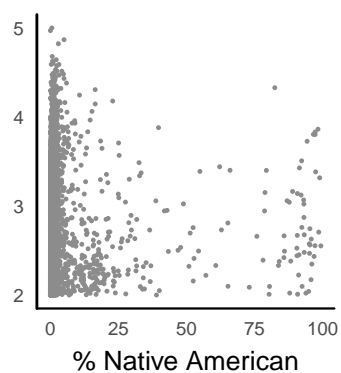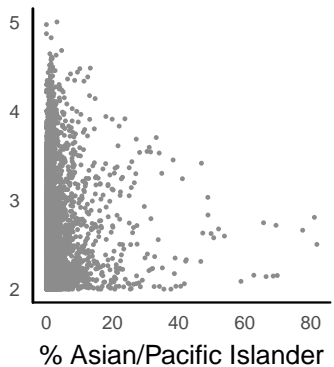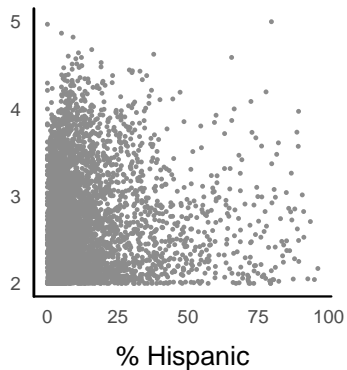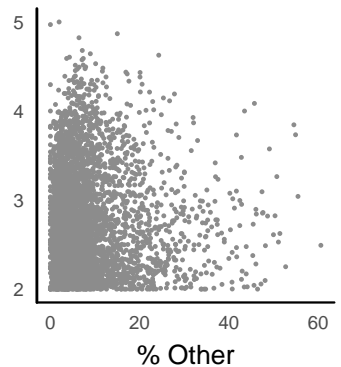

Supplement: S3 Fig — (PDF) [file pone.0205825.s003.pdf]

Resilience Index

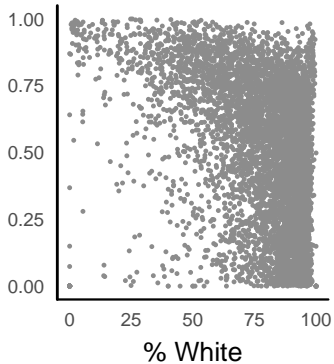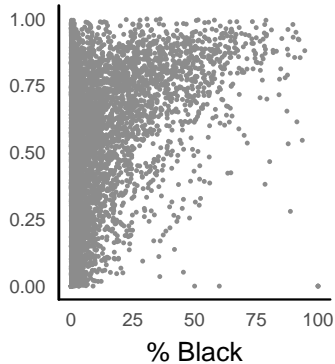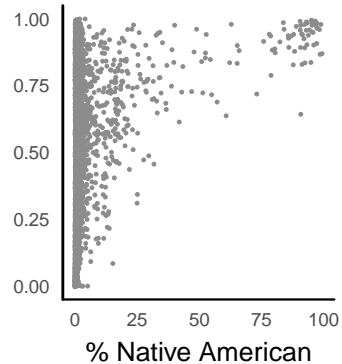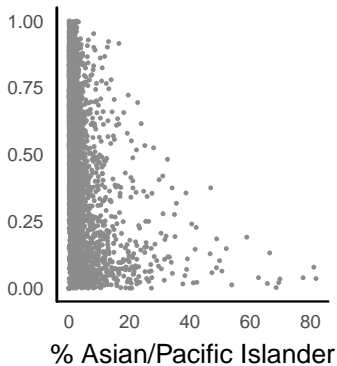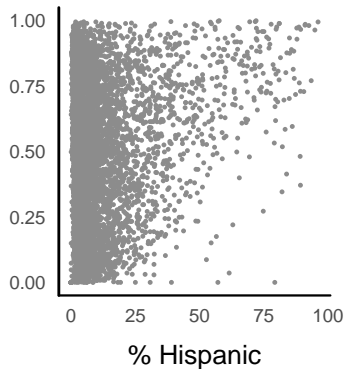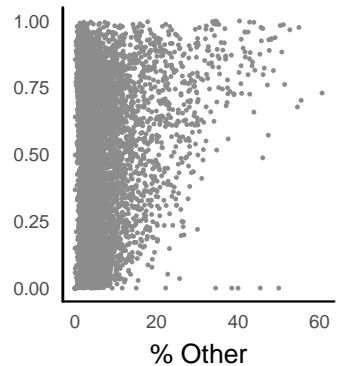

Supplement: S4 Fig — (PDF) [file pone.0205825.s004.pdf]
