# Supplementary material for: The unequal vulnerability of communities of color to wildfire
Source: PLoS One. 2018 Nov 2;13(11):e0205825. doi: 10.1371/journal.pone.0205825 (PMC6214520; doi:10.1371/journal.pone.0205825)
Supplement: S1 Table — Variables adapted from the Social Vulnerability Index using data from the American Community Survey 2000–2014 5-year estimates. (DOCX) [file pone.0205825.s005.docx]

| Domain | Variable |
| --- | --- |
| Socioeconomic Status | - Persons below poverty level - Civilians (age 16+) unemployed - Per capita income |
| Language and Education | - Persons (age 25+) w/out high school diploma - Persons (age 5+) who speak English “less than well” |
| Housing and Transportation | - Housing in structures w/ 10+ units - Mobile homes - Households with >1 persons per room - Households with no vehicle available - Persons in institutionalized group quarters |
| Demographics | - Civilian noninstitutionalized population w/ disability - Single parent household w/ children under 18 - Persons age 65+ - Persons age 17- |
