# Supplementary material for: The unequal vulnerability of communities of color to wildfire
Source: PLoS One. 2018 Nov 2;13(11):e0205825. doi: 10.1371/journal.pone.0205825 (PMC6214520; doi:10.1371/journal.pone.0205825)
Supplement: S3 Table — (DOCX) [file pone.0205825.s007.docx]

| White | Black | Native American | Asian/Pacific Islander | Hispanic | Other |
| --- | --- | --- | --- | --- | --- |
| -0.313 | 0.330 | 0.126 | -0.153 | 0.340 | 0.354 |
