# Supplementary material for: The unequal vulnerability of communities of color to wildfire
Source: PLoS One. 2018 Nov 2;13(11):e0205825. doi: 10.1371/journal.pone.0205825 (PMC6214520; doi:10.1371/journal.pone.0205825)
Supplement: S4 Table — A greater β at the 0.5 vulnerability quantile indicates that increases in the share of Hispanics, Blacks, Native Americans, and Other races is associated with a greater increase in vulnerability for the least vulnerable census tracts than for the most vulnerable tracts. In other words, increasing the population of these groups leads to more dramatic jumps in vulnerability for initially less vulnerable tracts. (DOCX) [file pone.0205825.s008.docx]

|  | Race/Ethnicity | | | | | | |
| --- | --- | --- | --- | --- | --- | --- | --- |
| Quantile | Value | White | Black | Hispanic | Native American | Asian / Pacific Islander | Other |
| 0.05 | β | -0.200 | 0.418 | 0.279 | 0.570 | -0.753 | 0.710 |
|  | SE | 0.023 | 0.043 | 0.034 | 0.031 | 0.038 | 0.061 |
| 0.95 | β | -0.267 | 0.113 | 0.135 | 0.212 | -0.779 | 0.257 |
|  | SE | 0.010 | 0.006 | 0.021 | 0.027 | 0.133 | 0.042 |
